# Subcellular localization of enzymes involved in the biosynthesis of digoxin in *Digitalis lanata*

**DOI:** 10.3389/fpls.2026.1703671

**Published:** 2026-02-27

**Authors:** Viviane Zeng, Emily Carroll, Devon Burnside, Zhen Q. Wang

**Affiliations:** Department of Biological Sciences, University at Buffalo, The State University of New York, Buffalo, Buffalo, NY, United States

**Keywords:** 3*β*HSD, cardenolides, CYP11A1, CYP87A, cytochrome P450, P450_scc_, P5*β*R2

## Abstract

Digoxin is a cardiac glycoside derived from the foxglove plant *Digitalis lanata* and is widely prescribed for treating heart failure and atrial fibrillation. Despite its medical importance, the cardiac glycoside biosynthetic pathway is only partially understood. Furthermore, the subcellular localizations of the three known enzymes for digoxin biosynthesis have escaped investigation. In this study, we identified the subcellular localization of the three known enzymes in the digoxin biosynthetic pathway, including the cytochrome P450 sterol side-chain cleaving enzyme (P450_scc_), the 3*β*-hydroxysteroid dehydrogenase (3*β*HSD), and the progesterone-5*β*-reductase 2 (P5*β*R2). Expressing these enzymes with a fluorescent tag in tobacco leaves revealed that the P450_scc_ localized to the endoplasmic reticulum (ER), and the 3*β*HSD and P5*β*R2 localized to the cytosol. The ER-localization of the foxglove P450_scc_ is of particular interest because the mammalian P450_scc_, or CYP11A1, is localized to the mitochondria. These findings provide key insights into the spatial organization of digoxin biosynthesis and guide future synthetic biology and metabolic engineering of digoxin biosynthesis in plants and microbes.

## Introduction

The foxglove plant *Digitalis lanata* synthesizes specialized metabolites called cardiac glycosides (Kreis, 2017). Digoxin is a medicinally crucial cardiac glycoside for treating heart failure since 1954, when the Food and Drug Administration (FDA) approved it for clinical use (David and Shetty, 2024). Digoxin and other cardiac glycosides act by allosterically inhibiting the Na^+^/K^+^ ATPase ion channel of heart muscle cells, which indirectly elevates the intracellular Ca^2+^ level, leading to increased cardiomyocytes’ contractile force (Smith et al., 1984). Structurally, cardiac glycosides consist of a steroid-like aglycone attached to an oligosaccharide moiety via a *β*-1,3-glycosidic linkage (Ravi et al., 2020). Despite the long-standing clinical applications, how foxglove and other plants synthesize digoxin and cardiac glycosides is only partially known. Only a few enzymes responsible for cardiac glycoside biosynthesis have been known so far.

**Figure 1:**
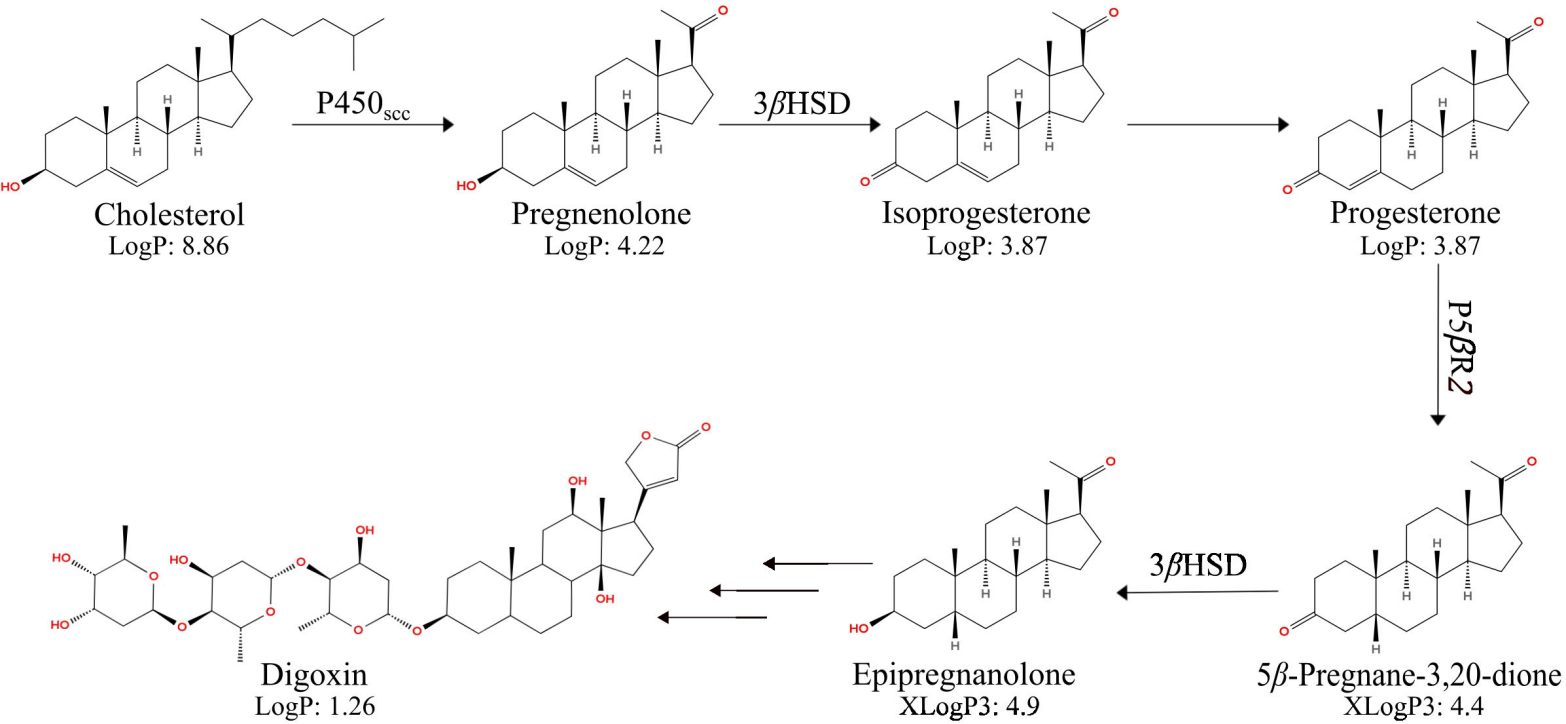
The biosynthetic pathway of digoxin in *D. lanata*. Besides cholesterol, campesterol and sitosterol can also serve as substrates for P450_scc_. P450_scc_: cytochrome P450 steroid side-chain cleaving enzyme; 3*β*HSD, 3*β*-hydroxysteroid dehydrogenase; P5*β*R2, progesterone-5*β*-reductase 2. LogP: Experimentally determined octanol-water partition coefficients retrieved from the Human Metabolome Database and literature search (Fornasier et al., 2020; Hansch and Hoekman, 1995; de Pádua et al., 2022). XLogP3: *In silico* estimated octanol-water partition coefficients (neutral molecular form) computed using XLogP3 3.0 (Cheng et al., 2007). These computational estimates provide relative hydrophobicity comparisons and do not represent experimentally measured values.

Recent works revealed three enzymes involved in the early steps of cardiac glycoside biosynthesis in foxglove plants. The cytochrome P450 sterol side-chain cleaving enzyme (P450_scc_) converts cholesterol, campesterol, or sitosterol to pregnenolone (Carroll et al., 2023; Kunert et al., 2023; Younkin et al., 2024). The 3*β*-hydroxysteroid dehydrogenase (3*β*HSD) oxidizes pregnenolone to isoprogesterone, which isomerizes into progesterone. The progesterone-5*β*-reductase 2 (P5*β*R2) reduces progesterone to 5*β*-pregnane-3,20-dione with regio- and stereospecificity (Herl et al., 2006; Pérez-Bermúdez et al., 2010). The 3*β*HSD further reduces 5*β*-pregnane-3,20-dione to epipregnanolone (**Figure 1**).

Despite the recent biochemical characterization of these three enzymes, their subcellular localization is still unexplored. Understanding the subcellular localization of these enzymes is crucial for revealing the spatial organization of the cardiac glycoside biosynthesis in a plant cell. A clear picture of the subcellular localization of pathway enzymes can generate hypotheses for missing transporters. It will also facilitate synthetic biology and metabolic engineering to produce medicinally important cardiac glycosides in alternative hosts such as *S. cerevisiae* and *E. coli*. Since enzymes’ subcellular localization can influence their activities, substrate availability, and metabolite trafficking, targeting enzymes to the appropriate organelles is critical for bioengineering to ensure pathway efficiency (Srinivasan and Smolke, 2021; Yao et al., 2023).

To this end, we investigated the subcellular localization of P450_scc_, 3*β*HSD, and P5*β*R2 by transiently expressing green fluorescent protein (GFP)-tagged enzymes in *Nicotiana benthamiana*, followed by protoplast isolation and fluorescence microscopy. The results showed that the foxglove P450_scc_ localizes to the endoplasmic reticulum (ER), whereas 3*β*HSD and P5*β*R2 are cytosolic, agreeing with bioinformatic predictions.

## Method

### Plant materials

Five-to-six-week-old *Nicotiana benthamiana* plants were grown on the Miracle-Gro potting mix (Scotts Miracle-Gro, Marysville, OH) and under a light period of 16-h light and 8-h dark with a relative humidity of 50-70%.

### Subcellular localization prediction

The amino acid sequences of P450_scc_, 3*β*HSD, and P5*β*R2 from *Digitalis lanata* were analyzed using four prediction programs: DeepLoc 2.0, WoLF PSORT, Plant-mSubP, and TargetP 2.0 (Almagro Armenteros et al., 2019; Horton et al., 2007; Sahu et al., 2019; Thumuluri et al., 2022). All predictions were based on full-length sequences. TargetP 2.0 evaluates the presence of signal peptides (Almagro Armenteros et al., 2019). **Supplementary Table 1** lists the gene sequences of *P450_scc_, 3βHSD*, and *P5βR2*.

### Gene constructs and cloning

#### P450_scc_, 3βHSD, P5βR2

Polymerase chain reaction (PCR) was performed for each gene using the Phusion high-fidelity DNA polymerase (New England Biolabs, Ipswich, MA). The PCR templates were the same genes previously cloned into a pYTK001 part plasmid (Lee et al., 2015). The PCR primers contained a BsaI site and a 4-nt Type 3 overhang on each end (Lee et al., 2015). A three-amino acid linker encoding “GSG” replaced the stop codon. Each gene was fused with the *GFP* gene at the C terminus and cloned into a pEAQ-MoClo plasmid using Golden Gate Cloning (Engler et al., 2009; Sainsbury et al., 2009). The C-terminal fused *GFP* gene was codon-optimized for expression in *Nicotiana benthamiana*. The resulting PCR product was transformed into the DH5α chemically competent *Escherichia coli* by heat shock at 42°C for 90 seconds. The colonies emerged the next day were screened by colony PCR and restriction digestions. The positive construct was sequence-verified by whole-plasmid sequencing (Plasmidsaurus, South San Francisco, CA). The sequence-verified constructs were transformed individually into the *Agrobacterium tumefaciens* strain AGL1 using the freeze-thaw method (Wise et al., 2006). **Supplementary Table 2** lists the primer sequences used in this work.

#### mScarlet, mScarlet-HDEL, GFP

The codon-optimized *mScarlet3-HDEL* for tobacco expression was synthesized by Twist Bioscience, South San Francisco, CA, and cloned into the pEAQ-MoClo plasmid by Golden Gate cloning (Engler et al., 2009; Gadella et al., 2023). HDEL is a four-amino-acid ER-targeting peptide (Gomord et al., 1997). To clone the cytosolic marker construct pEAQ_*mScarlet*, *mScarlet* was PCR amplified using the Phusion high-fidelity DNA polymerase (New England Biolabs, Ipswich, MA) with the synthetic *mScarlet3-HDEL* as the template, then cloned into the pEAQ-MoClo by Golden Gate cloning using the BsaI Type II restriction enzyme. The resulting colonies were screened by colony PCR and enzyme digestion, then followed by whole-plasmid sequencing (Plasmidsaurus, South San Francisco, CA). The *Aequorea victoria GFP (Q80R)* cytosolic marker construct pEAQ-HT-*GFP* was purchased from Leaf Systems International Ltd. **Supplementary Table 1** lists the gene sequences of *mScarlet3-HDEL* and *GFP*. Three plasmids were transformed into *A. tumefaciens* strain AGL1 individually by the freeze-thaw method (Wise et al., 2006).

### Tobacco transient expression

One *A. tumefaciens* colony transformed with one of a pEAQ plasmid bearing one of the three genes of interest was inoculated into 5 mL of Luria-Bertani (LB) broth with 50 mg/L kanamycin for the pEAQ plasmid selection and 25 mg/L rifampicin for the *A. tumefaciens* strain selection. The bacterial cultures were grown at 28°C with shaking at 220 rpm for 24 hours. The cultures were centrifuged at 3,000 x g for 10 min, and washed with 10 mL of sterile double-distilled water. The pellet was then resuspended in 2 mL of MMA solution [10 mM MES (2-N-morpholinoethanesulfonic acid), pH 5.6, 10 mM MgCl_2_, 100 μM acetosyringone] (Saxena et al., 2016). The O.D.600 of each *Agrobacterium* suspension was recorded by a UV-vis spectrophotometer. A 1:1 mixture of one *A. tumefaciens* strain containing the GFP-tagged gene of interest and another strain expressing the cellular localization marker was prepared. The mixture had a final volume of 10 mL, and each strain had a final OD_600_ of 0.4 in this mixture. This mixture was then incubated at room temperature for 2 to 4 hours and then infiltrated into the underside of five-to six-week-old *Nicotiana benthamiana* leaves with a needleless syringe. The tobacco plants were grown in a 16-h light and 8-h dark period for 2 days under dark condition for an additional 3 days to decrease the number of chloroplasts in the cell.

### Protoplast and imaging

Protoplasts from the transgenic tobacco leaves were prepared as previously described (Yoo et al., 2007). Briefly, seven to ten infiltrated tobacco leaves were cut into 1 mm-wide strips with a razor blade and incubated in 10 mL enzyme solution [20 mM MES, pH 5.7, 1.5% cellulase R-10 (GoldBio, St. Louis, MO), 0.4% macroenzyme R-10 (GoldBio, St. Louis, MO), 0.4 M mannitol, 20 mM KCl, 10mM CaCl_2_, 0.1% bovine serum albumin] followed by 20 min of vacuum infiltration. After incubation for two to three hours at room temperature,10 ml of W5 solution (2 mM MES, pH 5.7, 154 mM NaCl, 125 mM CaCl_2_, 5 mM KCl) was added to the mixture. The protoplasts were filtered using a 75nm nylon mesh and settled by gravity for 5 min in a round-bottom culture tube at room temperature. The clear supernatant was then discarded, and 2 μL of the settled protoplast pellet was loaded to the center of a glass slide and covered with a cover slip for imaging. Localization experiments were performed with at least three independent biological replicates (separate agroinfiltration events).

We used a Leica DMi8 fluorescence microscope for imaging. The excitation/emission wavelengths are 475 nm/510 nm for GFP; 560 nm/590 nm for mScarlet3, and 635 nm/700 nm for chloroplasts. The images were pseudo-colored as green for GFP-tagged enzymes of interest, red for the cytosolic or the ER control, and cyan for chloroplast autofluorescence.

## Results

### Prediction of subcellular localizations

We utilized four online prediction tools, DeepLoc2.0, WoLFPSORT, Plant-mSubP, and TargetP2.0, to predict the subcellular localization of the enzymes P450_scc_, 3*β*HSD, and P5*β*R2 (Almagro Armenteros et al., 2019; Horton et al., 2007; Sahu et al., 2019; Thumuluri et al., 2022) (**Table 1**). All four programs consistently predicted P450_scc_ to localize to the ER. In contrast, predictions for 3*β*HSD and P5*β*R2 varied: both DeepLoc2.0 and WoLFPSORT predicted them as cytosolic, while Plant-mSubP predicted 3*β*HSD to be in the plastid and P5*β*R2 to the cell wall, albeit with low confidence.

**Table 1:** Predicted subcellular localizations of P450_scc_, 3*β*HSD, and P5*β*R2.

Enzymes	DeepLoc 2.0	WoLF PSORT	Plant-mSubP	TargetP 2.0
P450_scc_	ER (93%)	ER (38%)	ER (67%)	Secretory pathway (69%)
3*β*HSD	Cytosol (55%)	Cytosol (64%)	Plastid (52%)	Not plastid or mt
P5*β*R2	Cytosol (77%)	Cytosol (92%)	Cell Wall (59%)	Not plastid or mt

ER, endoplasmic reticulum; mt, mitochondrion.

### Subcellular localization of P450_scc_, 3*β*HSD, and P5*β*R2

Given these predictions, we experimentally determined the subcellular localization of P450_scc_, 3*β*HSD, and P5*β*R2 by fusing a GFP tag with each protein and transiently expressing the fusion enzymes in *Nicotiana benthamiana* leaves. We then generated protoplasts from these transgenic leaves and visualized the enzymes’ subcellular distribution using a fluorescence microscope. GFP was fused onto the C-terminus of each protein because DeepLoc 2.0 (Thumuluri et al., 2022) predicted the ER targeting peptide is the first 30 amino acids at the N-terminus of P450_scc_, thus N-terminal GFP fusion may compromise this enzyme’s localization. Since 3*β*HSD and P5*β*R2 have no predicted targeting peptides, fusing GFP to the C-termini will unlikely change their subcellular localizations. We placed a short flexible “GSG” linker between the target protein and the GFP. Each fusion protein was co-expressed with an ER marker, mScarlet3-HDEL, or a cytosolic marker, mScarlet3, in tobacco leaves (Gomord et al., 1997). **Supplementary Figure 1** validates the ER localization of the mScarlet3-HDEL marker and the cytosolic localization of the mScarlet3 marker.

**Figure 2:**
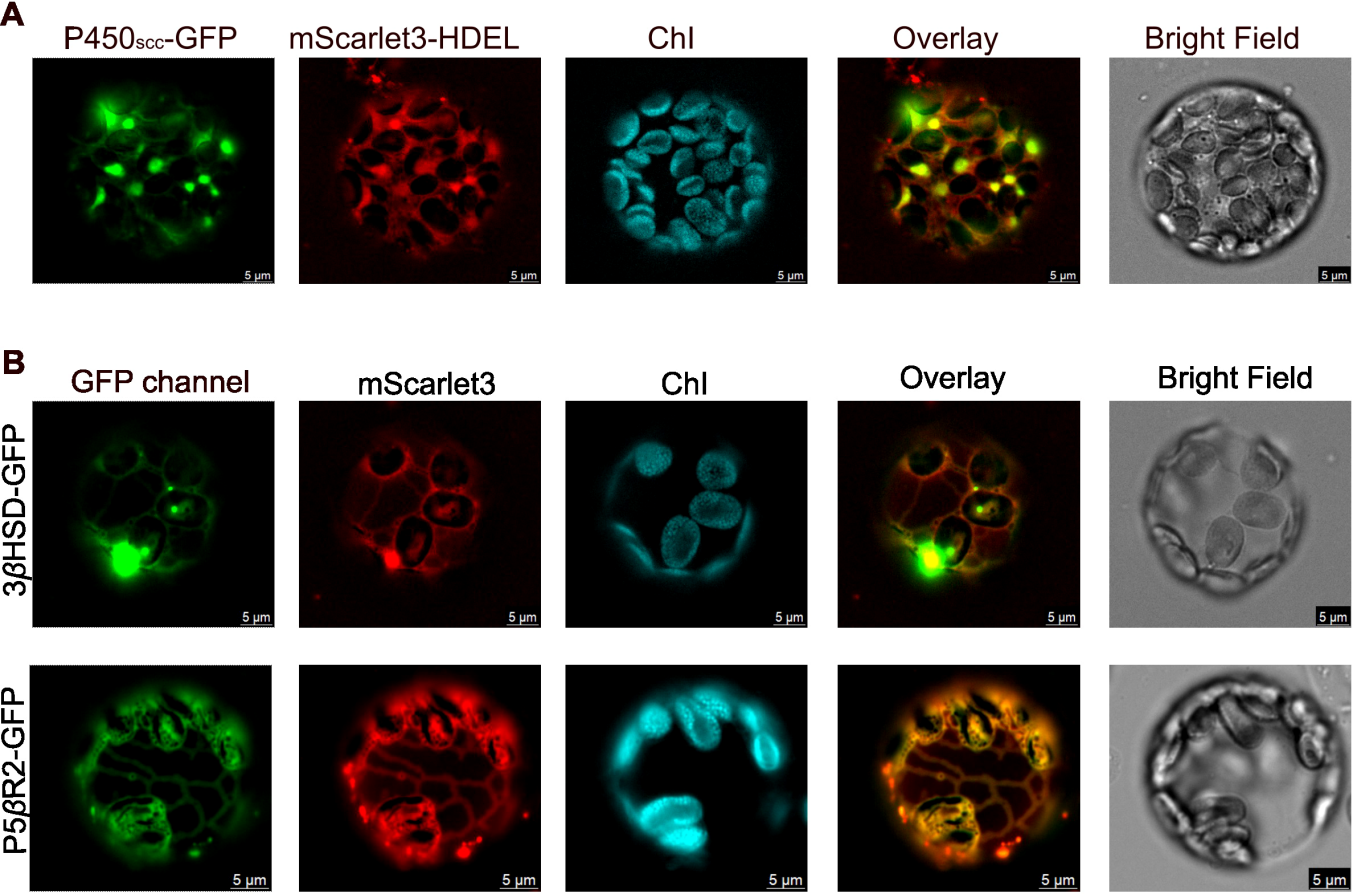
Fluorescence imaging of P450_scc_, 3*β*HSD, and P5*β*R2 fused with a green fluorescent protein (GFP) tag on the C-termini. **(A)** P450_scc_-GFP was co-expressed with an ER control, mScarlet3-HDEL, in tobacco protoplasts; **(B)** 3*β*HSD-GFP or P5*β*R2-GFP co-expressed with a cytosolic control, mScarlet3. ChI, Chloroplast autofluorescence; Overlay, the overlay images of the GFP channel and the mScarlet3 channel. Scale bar = 5 µm. Images are representative of consistent patterns observed across three independent biological experiments.

P450_scc_ co-localized with the ER marker as indicated by the overlay of the green fluorescence from P450_scc_-GFP and the red mScarlet3-HDEL marker (**Figure 2A**). Six out of seven protoplasts imaged showed the same ER localization. This result supports the predictions of DeepLoc2.0, WoLF PSORT, and Plant-mSubP, all of which identified P450_scc_ as an ER-localized enzyme. When co-expressed with the cytosolic marker mScarlet3 (**Supplementary Figure 2A**), P450_scc_-GFP displayed a reticular pattern distinct from the diffused cytosolic signal, further confirming its ER localization.

3*β*HSD and P5*β*R2 showed clear cytosolic distributions that overlapped with the mScarlet3 cytosolic marker (**Figure 2B**). Five out of the seven 3*β*HSD-expressing protoplasts and four out of the five P5*β*R2-expressing protoplasts exhibited the same cytosolic localization. These observations support the predictions of DeepLoc2.0 and WoLF PSORT, but not Plant mSub-P. Conversely, when co-expressed with the ER marker mScarlet3-HDEL (**Supplementary Figure 2B**), 3*β*HSD and P5*β*R2 exhibited no fluorescence overlap, further supporting that 3*β*HSD and P5*β*R2 are not on the ER but in the cytosol.

## Discussion

In this study, we experimentally determined the subcellular localizations of key enzymes involved in digoxin biosynthesis, including *Digitalis lanata* P450_scc_, 3*β*HSD, and P5*β*R2, using tobacco transient expression. Results confirm the ER localization of P450_scc_ and the cytosolic localization of 3*β*HSD and P5*β*R2. Although tobacco transient expression is a standard technique to investigate proteins’ subcellular localization, limitations of this technique, such as using a strong constitutive CaMV 35S promoter, fluorescent tagging, potentially altered protein-protein interactions, may affect the localization pattern of proteins of interest. However, it is less likely in this study since the tobacco leaves expressing the three GFP-tagged proteins still produced the expected pathway intermediates at similar levels as the untagged wildtype proteins, as shown in **Supplementary Figure 3**. In other words, the C-terminal GFP-tagged P450_scc_, 3*β*HSD, and P5*β*R2 remained functional. While transient expression in *N. benthamiana* provides a valuable system for initial localization studies, future work should confirm these localizations in native *D. lanata* tissues.

*Digitalis* P450_scc_, which belongs to the CYP87A family, catalyzes the first and rate-limiting step in digoxin and cardiac glycoside biosynthesis: cleaving cholesterol and phytosterols to pregnenolone (Carroll et al., 2023; Kunert et al., 2023; Younkin et al., 2024). The mammalian P450_scc_, or CYP11A1, also cleaves cholesterol to pregnenolone for synthesizing steroidal hormones (Strushkevich et al., 2011). However, the latter is localized to the mitochondria, whereas the former is in the ER, as determined in this work. This difference in subcellular localization, along with their low similarity in protein sequences, further supports that the plant and animal P450_scc_ are evolved independently via convergent evolution (Carroll et al., 2023). The ER-localization of *Digitalis* P450_scc_ also implies that it accepts electrons from an ER-localized cytochrome P450 reductase (Carroll et al., 2023). Such reductase has been well-studied in *Arabidopsis thaliana*, for example ATR2, which contains both FAD and FMN as cofactors (Niu et al., 2017). In contrast, the mammalian CYP11A1 relies on the mitochondrial FAD-containing adrenodoxin reductase AdR and the [2Fe-2S]-containing adrenodoxin Adx to transfer electrons from NADPH to the CYP450 (Shang and Huang, 2020; Strushkevich et al., 2011).

**Figure 3:**
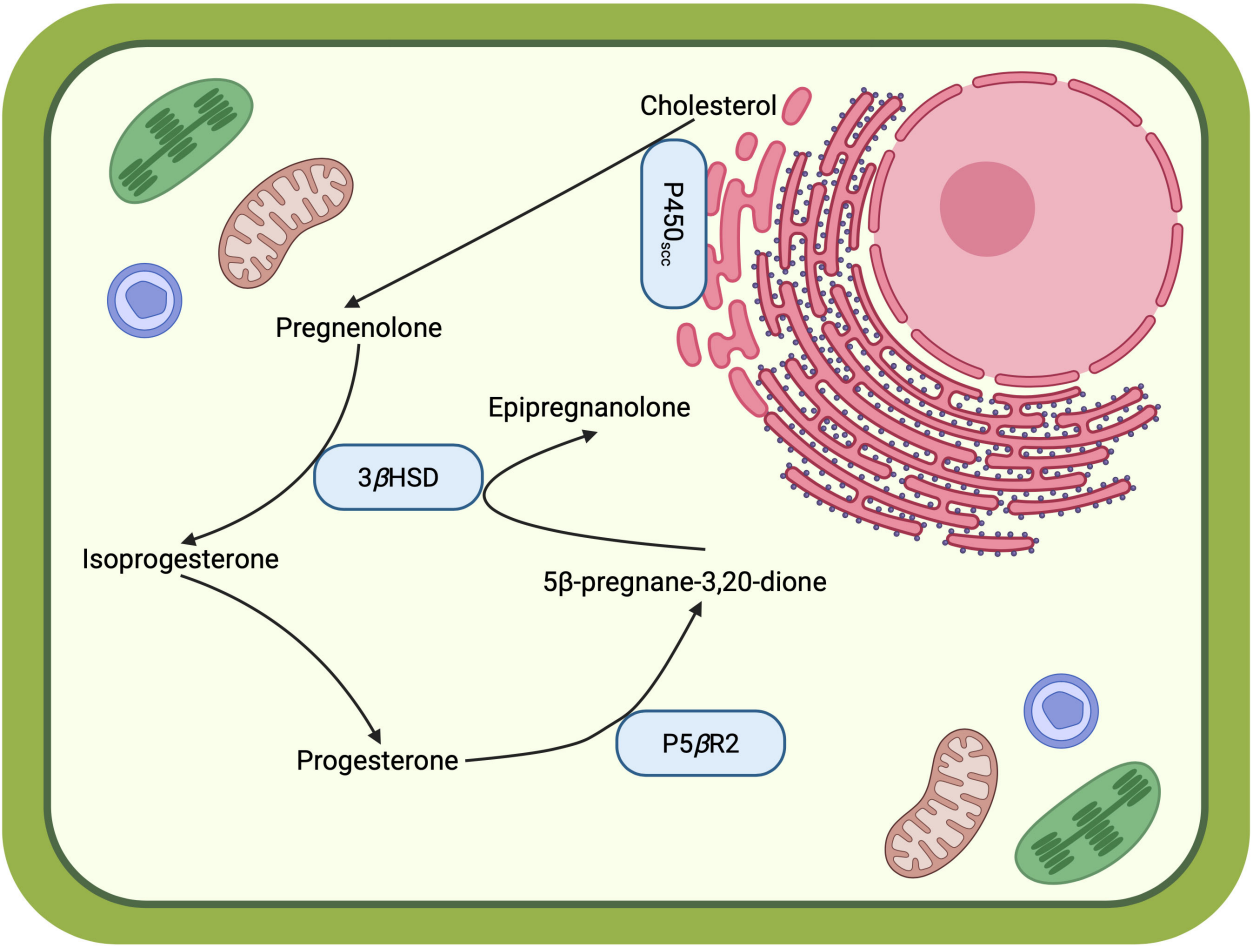
Working model of the subcellular organization of early digoxin biosynthetic steps. Solid arrows represent enzymatic reactions with experimentally determined protein localization. This model is based on the experimental data presented in this study but requires further validation for small molecule transport. This figure is created in BioRender.

In the current study, we begin to map the little-studied subcellular organization of digoxin biosynthetic enzymes (**Figure 3**). The ER-localized *Digitalis* P450_scc_ cleaves off the side chains of the cholesterol or phytosterols synthesized in the ER membrane, generating the more polar pregnenolone. Pregnenolone is then converted to other pregnane-intermediates by 3*β*HSD and P5*β*R2 in the cytosol for digoxin biosynthesis. Such hypothesis is supported by the hydrophobicity of these steroidal compounds, shown as experimentally determined or estimated octanol-water partition coefficients (logP) in **Figure 1**. Although pregnenolone is almost insoluble in water, it may be soluble in the hydrogel-like cytosol crowded with macromolecules (Fels et al., 2009). While our data establish the subcellular compartments where key reactions occur, the mechanism by which hydrophobic intermediates, such as pregnenolone, move between the ER membrane and cytosol remains to be determined. Future studies should investigate potential lipid transfer proteins or membrane contact sites that might facilitate this transport. Taken together, the spatial organization of the cardiac glycoside biosynthetic pathway revealed in this work will facilitate metabolic engineering to produce these medicinally crucial molecules in heterologous hosts.

## Data Availability

The datasets presented in this study can be found in online repositories. The gene sequence can be found in the article/**Supplementary Material**.
