# Supplementary material for: Subcellular localization of enzymes involved in the biosynthesis of digoxin in Digitalis lanata
Source: Front Plant Sci. 2026 Feb 27;17:1703671. doi: 10.3389/fpls.2026.1703671 (PMC13011968; doi:10.3389/fpls.2026.1703671)
Supplement: Supplementary file 1 [file DataSheet1.pdf]

Supplemental Information for

Subcellular Localization of Enzymes Involved in the Biosynthesis of Digoxin in *Digitalis lanata*

Viviane Zeng[1], Emily Carroll[1], Devon Burnside[1], Zhen Q. Wang[*1]

1. Department of Biological Sciences, University at Buffalo, the State University of New York, Buffalo, Buffalo, NY14260, United States

**Corresponding author: Zhen Q. Wang (zhenw@buffalo.edu), 653 Cooke Hall, Department of Biological Sciences, University at Buffalo, Buffalo, NY14260*

Supplemental Method

LC/MS analysis for pregnane intermediates

Tobacco leaves were infiltrated with *A. tumefaciens* at an O.D. of 0.4 for each strain transformed with a pEAQ plasmid carrying a gene of interest, as described in the Method section of the manuscript. Plants were grown in 16-hours light and 8-hours dark cycles. At day six, three ~2 cm leaf disks from three separate leaves on the same plant were harvested into three separate Eppendorf tube. The samples were flash-frozen in liquid nitrogen, ground, and 1 mL of 80% methanol was added immediately followed by vortexing for 1 minute. The samples were heated at 60°C for 10 minutes, centrifuged at 17,000g for 10 minutes, and finally filtered through a 0.45 µm syringe filter into a GC vial. The prepared samples were then subjected to LC-MS^2 analysis as previously described[1]. Briefly, a Waters XSelect CSH™ C18 HPLC column (particle size 3.5 µm, I.D. 2.1 mm, length 150 mm) was installed onto the Thermo Scientific Q-Exactive Focus™. Mobile phase A consisted of water and 0.1% formic acid. Mobile phase B contained acetonitrile and 0.1% formic acid. The flow rate was 200 µL/min, and the sample injection volume was 10 µL. The gradient started with 10% mobile phase B followed by a 12-min linear increase to 95% mobile phase B, then held for 2 min and brought back to the initial condition within 1 min. A full m/z scan with data-dependent MS^2 was used. The resolution of the first full m/z scan was 70,000, and the resolution of the second data-dependent MS scan was 17,500. Pure authentic standards were purchased from commercial sources. The experiment was repeated with two individual *Agrobacterium* infiltration events.

Supplemental Data

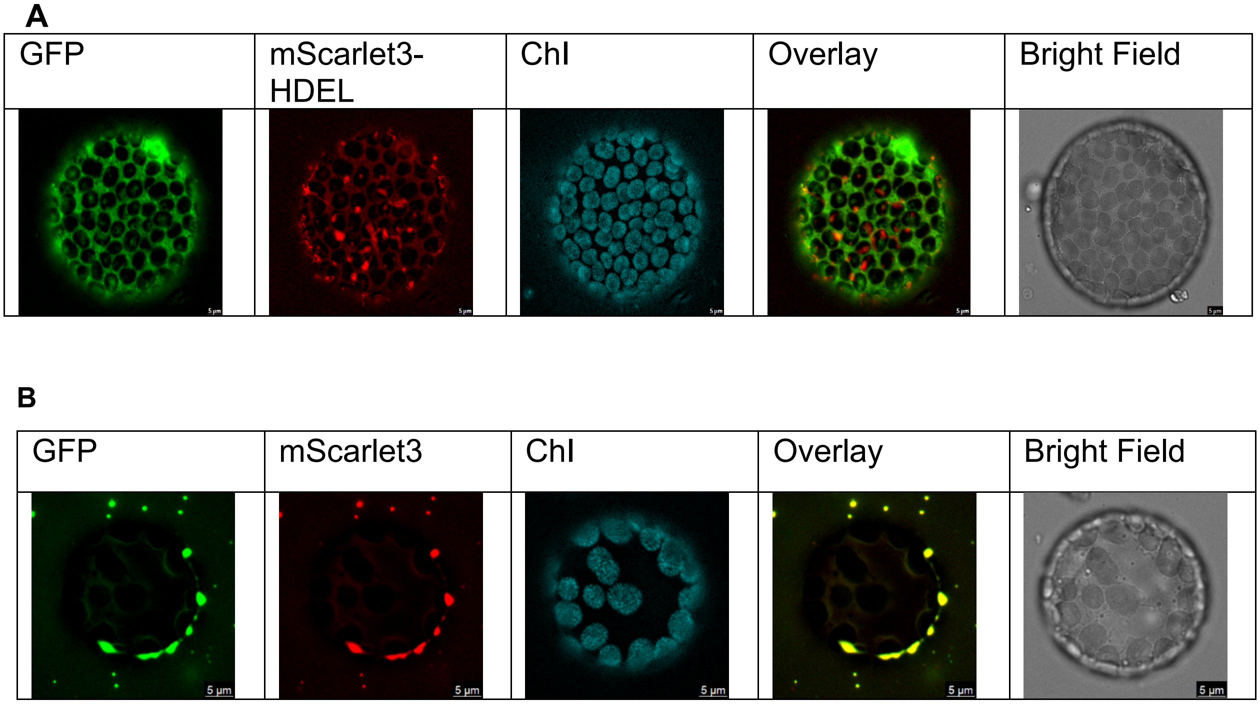

Supplemental Figure 1. Validating the ER and the cytosolic markers. Both the mScarlet3-HDEL (A) and the mScarlet3 (B) were co-expressed with GFP, a well-known cytosolic marker. mScarlet3-HDEL is primarily ER-localized (A), and mScarlet3 is cytosolic (B). Chl: Chloroplast autofluorescence; Overlay: the overlay images of the GFP channel and the mScarlet3 channel. Scale bar = 5 µm. Images are representative of consistent patterns observed across three independent biological experiments.

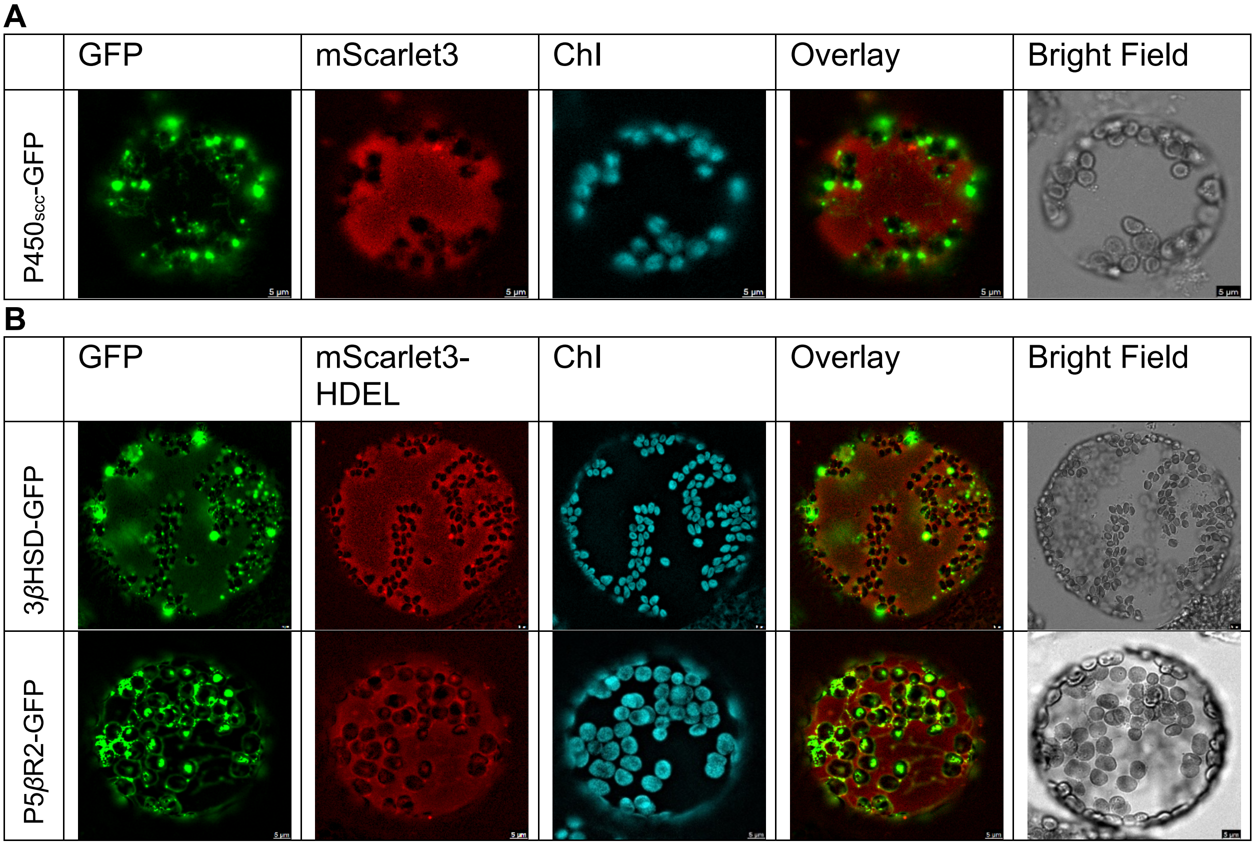

Supplemental Figure 2. Confocal fluorescent images of $P450_{scc}$-GFP, 3*β*HSD-GFP and P5*β*R2-GFP with markers opposite of their expected subcellular localization. (A) $P450_{scc}$ was co-expressed with a cytosolic marker mScarlet3; (B) 3*β*HSD and P5*β*R2 were co-expressed with an ER marker mScarlet3-HDEL. Chl: Chloroplast autofluorescence; Overlay: the overlay images of the GFP channel and the mScarlet3 channel. Scale bar = 5 μm. Images are representative of consistent patterns observed across three independent biological experiments.

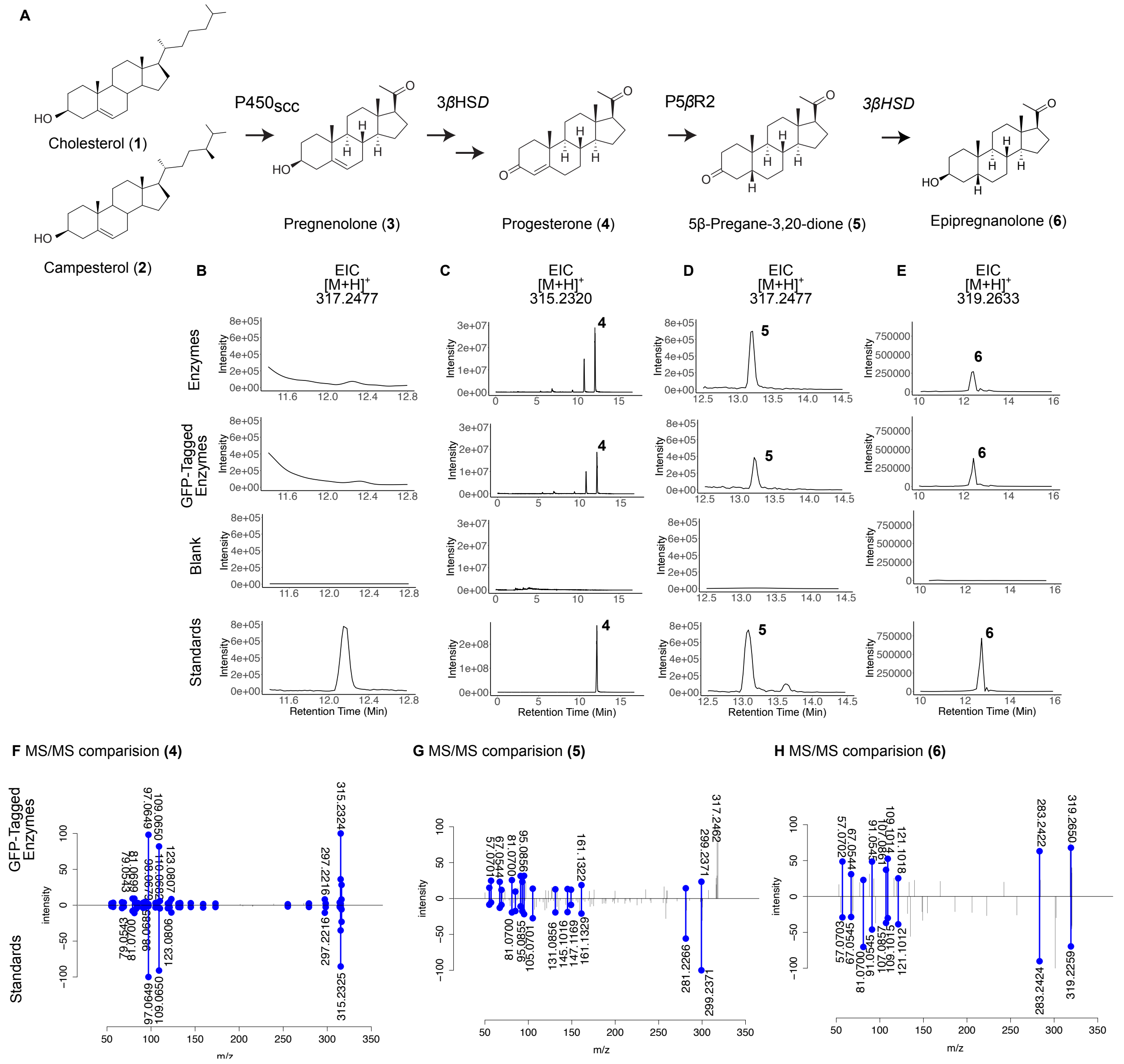

Supplemental Figure 3. Tobacco expression of $P450_{SCC}$, 3βHSD, and P5βR2 with or without GFP tags. (A) Schema of the upstream pathway of digoxin biosynthesis. (B-E) Extracted ion chromatograms (EIC) for pathway intermediates, including pregnenolone (compound 3), progesterone (compound 4), 5β-pregnane-3,20-dione (compound 5), and epipregnanolone (compound 6). The following experimental sets were included: Enzymes set – tobacco transiently expressing the three enzymes without GFP tags; GFP-tagged enzymes: tobacco transiently expressing the three enzymes with C-terminal GFP tags; Blank: tobacco expressing GFP only; Standards: authentic standards of pure chemicals. Chromatograms are representative of three leaf disks from separate leaves. (F-H) MS^2 spectra comparing the peaks from the GFP-Tagged enzyme set (top) to the authentic standards (bottom) for compounds 4, 5, and 6, respectively. Blue lines indicate peaks in the spectra that are within m/z 5ppm of the peaks from the standard.

Supplemental Table 1. Gene sequences of *mScarlet3-HDEL*, *$P450_{scc}$*, *3βHSD, P5βR2*, and *GFP*. *mScarlet3-HDEL* was codon-optimized for tobacco expression.

mScarlet3-HDEL	ATGATGGACAGTACCGAAGCCGTAATTAAGGAATTCATGCGATTTAAGGTACACATGG<br>AAGGATCAATGAACGGACACGAGTTCGAAATAGAGGGTGAAGGCGAGGGTCGTCC<br>GTATGAAGGGACACAAACTGCGAAACTAAGGGTCACCAAGGGCGGACCTTTGCCTT<br>TCTCATGGGATATCCTCAGCCCTCAATTTATGTATGGTTCTAGAGCATTTACTAAGCAC<br>CCAGCCGACATTCCAGACTATTGGAAGCAATCCTTTCCTGAGGGGTTCAAATGGGA<br>GCGAGTAATGAACTTTGAGGACGGTGGAGCTGTTAGTGTCGCTCAAGACACAAGCC<br>TGGAAGATGGCACACTTATATACAAAGTGAAATTACGTGGTACTAACTTTCCACCTGA<br>CGGACCTGTTATGCAAAAGAAGACCATGGGGTGGGAAGCCAGCACGGAGAGACTT<br>TACCCAGAAGACGTCGTCTTAAAGGGTGATATAAAGATGGCTCTCAGGCTTAAGGAT<br>GGTGGTAGATATTTAGCTGACTTCAAGACTACTTATCGTGCTAAGAAGCCTGTACAGA<br>TGCCCGGAGCATTTAACATAGATAGGAAACTAGACATCACATCTCATAACGAAGACTA<br>CACCGTGGTTGAGCAATACGAACGTAGTGTCGCTCGCCACTCTACCGGTGGAAGTG<br>GTGGCTCCGGATCCGGACACGACGAACTTTGA
$P450_{scc}$	ATGTCGTTAGTAGCTATGAGCGTTGGTGCTATTCTGATCATCATCACCATCATCACAA<br>ACTTGGTTTTTAAATGGTGGAACCGTTCCCTGAGCAGTGGAGGAGTGCTGCCTCCC<br>GGTTCATTCGGGTGGCCACTCATCGGAGAGACTCTTCACTTCTTCACTCCAAACAC<br>CTCATTCGATGTTACTCCTTTTGTAAAGGACAGAATGAAAAGATATGGGCCGATATTC<br>AAGACCAGCTTGGTGGGCGTTCCGGTGATAGTGTCGACTGACGCAGAGCTAAACAA<br>TTTCATCTTTCAGCAAGAGGGACAAACGTTTCAGAGCTGGTACCCAAGTACCTTCAC<br>TGAAATATTTGGCAGAGAAAATCTTAGTACTCTGCATGGCTTCATGTACAAGTACTTTA<br>AGAACATGGTACTAGGTTTATTTGGTCCCGAAAGCCTCAAAACTATGATTTCTGAGGT<br>TGAGAACACATCAAATATCAACTTAAAAAGGTGGTCGTCGTCCAATGGAACTGTCGA<br>ACTCAAGGATGCTATTGCAGAGATGATTTTCGAGCTGACTGCGAAGAAGCTAATCAG<br>TTATGAATTAGAAAAATCCCCTTACAATCTGAGGGATAATTTTGTGGCGTTCATAGATG<br>GGTTGATCAGCTTTCCTCTCAACATTCCGGGAACAGCCTATTACAGATGCTTACAGG<br>GAAGAAAAAATGCGATCAAGATGTTGAAAGATATGCTACATGAGAGGCGGGAAAAGC<br>CACGTGAAACACAAACCGACTTCTTTGACTATGTTCTAGAAGAACTTCAGAAAGAGG<br>ACACCATAATCACAGAGACACTTGCACTGGACTTGATGTTTGTGCTGCTCTTCGCCA<br>GCCACGAGACAGCTTCCATCGCCCTAACTTTGGCTATGAAATTTCTCGTCGACCATC<br>CCTTAGTGTTGGAAAAATTGACAGAAGAACACGACGAGATAATAAAAACCAGAGAAG<br>ATCCAAATTCAGGACTAACCTGGAACGAGTACAAATCAATGAAATTCACATTTCAGTT<br>CATCAATGAAACTCTAAGACTGGCAAATATAGCTCCTCTTATTTTCAGAAAAGCTCTC<br>ACTGAAACCGAATTTAAAGGATACACAATTCCAGCTGGTTGGGCTGTTATGGTATGTC<br>TTCCTGCTGTCCATTTAGACCCATCCAAATATAAAAATCCTCTCGAGTTTAATCCATGG<br>AGATGGGAGGGAGTAGATACAAGTGTTGGTTCAAAAACGTTCATGGCGTTCGGAGG<br>TGGAATGCGATTATGCATTGGGGCAGATTTCACAAAGGTCCAGATGGCTGTTTTCCT<br>CCATTGTTTGGTGACCAAGTACAAGTGGAAATCCATCAAAGGAGGAGACATTGTCCG<br>ATGCCCTGGCTTGAAATTCCCAAATGGATTTCACGTTAACATGACTGAGAGAGGC

3βHSD	ATGTCGTCAAAGCCAAGGTTGGAGGGTAAAGTGGCAATCATCACCGGAGCCGCTAG<br>CGGCATCGGCGAGGAGACGGCAAGATTGTTCGTGGAGCATGGCGCCTCAGTGGTG<br>GTGGCGGACGTCCAGGACGAATTGGGGCGCCAGGTCGTCGCTTCCGTAAACTCTG<br>ACGACAAGATAAGTTACTACCACTGCGATGTCAGAGATGAAAAACAAGTGGCGGCC<br>ACCGTCCGCTACGCGGTGGAGAAATACGGGCGCCTCGACATCATGCTGAGCAACG<br>CCGGAGTCTTCGGGGCCTTGATGACGAACGTAATCGATCTCGACATGGTTGACTTT<br>GAAAATGTATTGGCGACTAACGTGCGCGGAGTTGCCAACACTATAAAGCACGCGGC<br>ACGAGCCATGGTGGAGGGGAAGGTCAAGGGGTCCATCATTTGCACCGCCAGCGTG<br>TCGGCTAGCCTTGGAGGCATGGGCCCGCCCGCTTACACGGCTTCCAAACACGCCG<br>TCCTGGGCCTAGTCAAGGCGGCTTGCGCCGAGTTGGGGGTGCACGGGATCCGAGT<br>CAACTCGGTGGCGCCGTACGGTGTGGCGACCCCGATGCCGTGCAGTGCTTACGGA<br>ATGACACCGAGTCAGATGGAGGAGGCCAATAACTCCAGGGCTAACTTGAAGGGGGT<br>GGTTTTGAAGGCTAAGCATGTAGCTGAGGCGGCTCTCTTCTTGGCTTCCGATGAGT<br>CGGCTTATGTCAGTGGGCAAAACTTGGCTGTCGACGGCGGCTTCACCGTCGTGCG<br>T
P5βR2	ATGTATACCGACACAACGACTTGGTGGTACAAAAGATCCATTGGCGATATTAAACAGA<br>AAAATGTCGAAACAAATGGTGTTGCTTTAAACTACAAGAGTGTCGCCCTCATAGTTG<br>GGGTCACCGGCATAGCCGGAAGCGGTCTGGCTGAAACTCTATCCATGTCCGACACG<br>CCAGGTGGACCGTGGAAAGTCTACGGGGTCGCGCGCCGCCCTTGTCCAGAGTGG<br>CTAGCCAAACTCCACGTCAGCTACATCCAATGTGACATCGGGTCAACTGATGACACG<br>TCAGCCAAATTGTCTCCACTTTCCGACATCACTCACATTTTCTACGTGTCCTGGACC<br>GGATCCGAAGATTGTGATAAGAACGCAATCATGTTCAAAAACATCCTCGATTCAGTCA<br>TCCCCAATGCTCCAAATCTCAAACACATTTCCCTCCAAACAGGAATCAAGCACTATTG<br>GGGCAACATGGTTGATGAAATGGACACCACTAATGTATCACATGATTGTCCCTTTAAT<br>GAATACATGCCTAGACTTAGACAACCCAATTTCTATTACAATCTTGAAGACCTACTCTA<br>TGAAGCCTGCAGGACACAAAACGGTGCTCTAACATGGACCGTCCATCGCCCCGCG<br>CTAATTTTTGGGTTCTCACCATGTAGTTTGATGAACATAGTCGCGACGCTAAGTGTTT<br>ATGCCGCGATTTGCAAATATGAGAACAAGCCATTGGTGTATACCGGGACACAAACGT<br>CGTGGAATTGTTTAGTGGATGCTGTGGATTCGGATTTGTTAGCGGAGCACTTGGTAT<br>GGGGTGCGATCAGCCCAAACGCGAAGAACCAAGCTTTCAATATCAACAATGGCGAC<br>GTTTTTAAATGGAAACATATATGGAAAGTGTTGGCGGAGCAGTTTGAGATTGAGTTTG<br>TGGGTTATGAAGGTAAGGAGCCGGTGTCTTTGGAGGGTCTCATGAAGGATAAGGAC<br>TCGGTATGGGACGAGATGGTGGAGAAATACGACCTTGTCCCCACGAAACTTAGGGA<br>CATAGCGGCATTTTGGTTTGCGGATGTGGCGTTTAGCATCGAGGGAGCGGTTTCGA<br>GCATGAACAAGAACAAGGAGTTTGGGTTCATGGGGTTCAGGGATAGCACCAAGTCT<br>TTTATATCTTCTGTTAACAAAGTGAGATCTTATAGATTTGTCCCT
GSG-AvGFP (Q80R)	GGGTCTGGAATGACAAGTAAGGGTGAGGAGTTGTTTACGGGCGTGGTACCTATATT<br>GGTGGAGCTCGACGGGGACGTGAACGGCCATAAGTTCTCAGTGTCAGGGGAAGGA<br>GAAGGGGACGCCACTTATGGCAAGCTAACTCTGAAGTTCATATGTACAACCGGTAAG<br>CTCCCGGTACCTTGGCCCACGCTGGTGACAACGTTTTCCTACGGAGTGCAGTGTTT<br>CTCCCGATATCCCGACCACATGAAGAGGCACGATTTCTTTAAATCAGCTATGCCTGA<br>GGGCTACGTTCAAGAGAGGACAATCTTCTTTAAAGACGATGGCAATTATAAAACTCG<br>AGCCGAGGTGAAATTCGAGGGAGACACTTTAGTGAACCGTATAGAACTGAAGGGAA<br>TCGACTTCAAAGAGGACGGCAATATCCTCGGGCATAAGTTAGAGTACAATTACAATTC<br>TCATAACGTGTATATTATGGCTGATAAGCAGAAGAACGGGATCAAGGTGAACTTTAAG<br>ATACGCCATAATATCGAGGACGGGTCAGTGCAGTTAGCTGATCACTACCAGCAGAAC<br>ACACCGATCGGGGACGGTCCAGTTTTACTCCCCGATAATCACTATCTTAGTACTCAG<br>TCAGCACTGAGCAAGGACCCAAATGAGAAAAGGGATCATATGGTATTACTCGAATTC<br>GTTACGGCAGCGGGAATCACTCACGGTATGGACGAGTTATATAAGTAG

Supplemental Table 2. List of primers used in this study. Forward and reverse primer sequences are provided for cloning *$P450_{scc}$*, *3βHSD*, *P5βR2* and *mScarlet3* into the pYTK001 vector and the pEAQ vector via Golden Gate assembly.

$P450_{scc}$	Forward	TTTGGTCTCATATGTCGTTAGTAGCTATGAGCG
	Reverse	AAAGGTCTCTACCCGCCTCTCTCAGTCATGTTAACG
3βHSD	Forward	TTTGGTCTCATATGTCGTCAAAGCCAAGGTT
	Reverse	AAAGGTCTCTACCCACGCACGACGGTGAAG
P5βR2	Forward	TTTGGGGTCTCATATGTATACCGACACAACGAC
	Reverse	AAAGGTCTCAACCCAGGGACAAATCTATAAGATCTCAC
mScarlet3	Forward	TTTCGTCTCATCGGGGTCTCATATGGACAGTACCGAAGCC
	Reverse	GAAGTGGTGGCTCCTGAATCCAGAGACCGACCTGAGACGTTT
GSG-GFP	Forward	TTTGGTCTCATGGGTCTGGAATGACTAGCAAAGGAGAAGAAC
	Reverse	AAAGGTCTCAGGATTTATTTGTATAGTTCATCCATGCCA
pEAQ for Colony PCR	Forward	TCTTCTTCTTGCTGATTGGTTCT
	Reverse	TGTGTCCTTGCTGAAGGGAC

Supplemental reference

1. Ravi, B. G., Guardian, M. G. E., Dickman, R. & Wang, Z. Q. (2020). High-resolution tandem mass spectrometry dataset reveals fragmentation patterns of cardiac glycosides in leaves of the foxglove plants. Data in Brief, 30, 105464.
